# High level of treatment failure with commonly used anthelmintics on Irish sheep farms

**DOI:** 10.1186/2046-0481-67-16

**Published:** 2014-08-03

**Authors:** Orla M Keane, Jason D Keegan, Barbara Good, Theo de Waal, June Fanning, Michael Gottstein, Mícheál Casey, Christine Hurley, Maresa Sheehan

**Affiliations:** 1 Animal & Bioscience Department, AGRIC, Teagasc, Grange, Dunsany, Co, Meath, Ireland; 2 School of Veterinary Medicine, University College Dublin, Belfield, Dublin 4, Ireland; 3 Animal & Bioscience Department, AGRIC, Teagasc Mellows Campus, Athenry, Co, Galway, Ireland; 4 Department of Agriculture, Food and the Marine, Agriculture House, Dublin 2, Ireland; 5 Teagasc, Codrum, Macroom, Co, Cork, Ireland; 6 Regional Veterinary Laboratories Division, Backweston, Co, Kildare, Ireland; 7 Kilkenny Regional Veterinary Laboratory, Hebron Road, Kilkenny, Co, Kilkenny, Ireland

**Keywords:** Sheep, Nematode, Anthelmintic Resistance, Anthelmintic Efficacy

## Abstract

**Background:**

In 2013 a Technology Adoption Program for sheep farmers was established to encourage the implementation of best management practices on sheep farms in Ireland. There were 4,500 participants in this programme in 2013. As part of this programme, farmers had the option to carry out a drench test to establish the efficacy of their anthelmintic treatment.

**Results:**

Flock faecal samples were collected before and after treatment administration and gastrointestinal nematode eggs enumerated. In total there were 1,893 participants in the task, however only 1,585 included both a pre- and post-treatment faecal sample. Of those, 1,308 provided information on the anthelmintic product that they used with 46%, 23% and 28% using a benzimidazole (BZ), levamisole (LEV) and macrocyclic lactone (ML) product respectively. The remaining farmers used a product inapplicable for inclusion in the task such as a flukicide or BZ/LEV combination product. Samples were included for analysis of drench efficacy if the pre-treatment flock egg count was ≥200 eggs per gram and the interval post-sampling was 10–14 days for BZ products, 4–7 days for LEV products and 14–18 days for ML products. These criteria reduced the number of valid tests to 369, 19.5% of all tests conducted. If the reduction post-treatment was ≥95% the treatment was considered effective. Only 51% of treatments were considered effective using this criterion. There was a significant difference in efficacy between the anthelmintic drug classes with BZ effective in only 30% of treatments, LEV effective in 52% of cases and ML effective in 76% of cases.

**Conclusions:**

Gastrointestinal nematode anthelmintic treatments, as practiced on Irish farms, have a high failure rate. There was a significant difference between the efficacies of the anthelmintic classes with BZ the least effective and ML the most effective.

## Background

Grazing sheep are continually exposed to gastrointestinal nematodes (GIN). GIN infection in lambs can cause a reduction in voluntary feed intake, a decrease in digestive efficiency and protein loss from the gastrointestinal tract due to tissue damage [1]. Consequently, GIN infection reduces growth rates in young lambs leading to ill-thrift and occasional death. For over 50 years, the administration of broad spectrum anthelmintics has been an essential component in controlling the negative impact of GIN in sheep. Currently, there are five anthelmintic classes available for the control of nematode infection in sheep although the two most recently licensed products, an amino-acetonitrile derivative (Zolvix, Novartis) and a spiroindole/macrocyclic lactone combination product (Startect, Zoetis) are prescription-only medicines in Ireland. Therefore, there are three commonly used modern broad spectrum anthelmintic classes available in Ireland, benzimidazole (BZ), levamisole (LEV) and macrocyclic lactone (ML) [2].

Regular treatment with anthelmintics, in the absence of strategies to delay the development of resistance, will favour the survival of resistant nematode species. In recent years, reports of anthelmintic resistance, and resistance to more than one anthelmintic class, are increasing worldwide [2-7]. Anthelmintic resistance is defined as the heritable ability of some nematodes to survive treatment with an anthelmintic at the recommended therapeutic dose level [8]. In order to be considered fully effective, an ovine anthelmintic treatment must result in a reduction of faecal egg count (FEC) of at least 95% with the lower confidence limit greater than 90% [9]. If only one of these criteria is met then resistance is suspected. A limited number of studies have examined the prevalence of resistance to the commonly used anthelmintics in Ireland but have involved relatively small numbers of farms [2,5,10,11]. The most recent study in the Republic of Ireland found evidence of BZ resistance on almost 90% of farms tested and evidence of LEV resistance on almost 40% of farms tested [2]. Resistance to ML was also suspected on 11% of the farms [2]. In Northern Ireland, evidence of resistance to BZ, LEV and ML was found in 81%, 14% and 57% respectively of flocks tested [5]. These studies indicate a significant level of anthelmintic resistance among sheep nematodes in Ireland.

A number of factors are considered to influence the rate at which anthelmintic resistance arises and spreads. These include inappropriate dosing (dosing too often or not administering the correct dose quantity), the proportion of nematodes *in refugia* and the movement of animals harbouring resistant nematode populations [12]. A recent survey of Irish lowland sheep producers found that there was considerable departure from best practice in anthelmintic administration, which may accelerate the development of anthelmintic resistance [13]. This is a threat to sustainable sheep production in Ireland and indicates the need for an improvement in the technical efficiency in nematode control practices.

In 2013 the Department of Agriculture Food and the Marine (DAFM), Ireland, established a Sheep Technology Adoption Programme (STAP). The aim of this programme is to increase profitability on Irish sheep farms by using discussion groups to encourage and enable the adoption of best management practices. STAP is a two year programme, and participants in 2013 were required to attend at least four discussion group meetings with agricultural facilitators or three discussion group meetings and one national event. Participants were also required to complete two technical tasks from a list of ten possible tasks. Among the STAP task options was a drench test. This task was designed to test the efficacy of anthelmintic treatment as practiced on Irish farms. The findings from this study are summarized in this communication.

## Methods

### Farm profile

Farmers must either have a minimum of 30 breeding ewes or have purchased a minimum of 100 lambs/hoggets for breeding within the previous two years in order to qualify for inclusion in STAP. STAP participants who were selling lambs to processors were also required to apply for membership of the Board Bia Lamb Quality Assurance Scheme. No other restrictions were placed on STAP participants and so participants represented all types of sheep or mixed sheep farmers from every county in Ireland.

### Sample collection

Farmers choosing to enroll for the drench test task were given a detailed set of instructions describing the sampling protocol. These instructions were issued to them by the discussion group facilitators and were also available to download on the DAFM website [14]. The task was completed between June 1st and October 4^th^ 2013. To conduct the drench test, fresh faecal samples were collected from a minimum of 15 lambs that had not been treated with an anthelmintic product in the previous six weeks. Lambs were to be placed in a clean pen. A minimum of ten faecal deposits (representing different lambs) were to be placed in separate transport containers. Samples were sent by mail to a DAFM approved testing laboratory as soon as possible after collection. It was advised to keep the samples refrigerated if it was not possible to post on the day of sampling. On the day of sample collection, it was advised to mark the group of lambs that were faecal sampled and lambs were dosed with an anthelmintic product of the farmer’s choice from BZ, LEV or ML classes of anthelmintics. It was advised to weigh the three heaviest lambs in the group being tested and treat to the weight of the heaviest lamb in accordance with the manufacturer’s recommendations. Farmers were instructed to calibrate the dosing gun beforehand and also to ensure that the anthelmintic product was in date and well mixed before administration. Post-treatment, 10 fresh faecal samples were collected from the same group of marked lambs and sent to an approved laboratory for testing. These were to be taken seven days post-treatment if a LEV product was used or 14 days post-treatment if a BZ or ML product was used.

### Faecal egg counting

Seven commercial laboratories were approved by DAFM to accept and test faecal samples mailed by farmers. The conditions of approval by DAFM included participation in proficiency testing conducted by Vetqas [15]. Vetqas is the independent, accredited, proficiency testing unit of the United Kingdom’s Animal Health and Veterinary Laboratories Agency (AHVLA). Laboratories were supplied with a detailed protocol on how to generate the composite samples. Briefly, for each group of lambs to be tested, composite faecal samples were prepared so that each individual animal sample contributed the same unit weight to the composite sample (~3 g per lamb). Faecal egg counts were carried out according to the modified McMaster method [16] with both chambers of the McMaster slide counted with a sensitivity of 50 eggs per gram of faeces. FEC for *Nematodirus* species (FEC_NEM_) and other trichostrongyle species (FEC_OT_) (excluding *Strongyloides papillosus*) were enumerated separately.

### Data management and analysis

At the end of each month, the DAFM approved laboratories submitted the results of all tests conducted that month to Kilkenny Regional Veterinary Laboratory. Data were subsequently entered in an Excel spreadsheet and checked for anomalies and corrected. Data were screened to exclude participants who did not (1) provide both a pre- and post-anthelmintic treatment sample (2) report the anthelmintic product used (3) use an appropriate product for the task (4) adhere to the correct post sampling time interval or (5) include sufficient information to calculate sampling interval. The criteria used to assess whether the drench was effective was based on WAAVP recommendations [9] and FEC_NEM_ and FEC_OT_ data were analysed separately. Data were included in the analysis if the composite samples had a pre-treatment egg count of ≥200 eggs per gram for FEC_NEM_ or FEC_OT_ and if the post treatment count was carried out 10–14, 4–7 or 14–18 days for BZ, LEV and ML products respectively. When the reduction in FEC post-treatment was less than 95%, the treatment was considered ineffective. Results are expressed as the percentage of successful treatment for each class of anthelmintic and differences between the efficacies of the drug classes were calculated using a *χ*^2^ test in SPSS version 20 software. In order to determine if treatment failure varied across the country, samples were grouped according to their NUTS (Nomenclature of Territorial Units for Statistics) level 3 regional authority. Geographical relationships with treatment failure were calculated by creating contingency tables for NUTS level and treatment failure for each anthelmintic drug class and for all classes combined. In order to determine if treatment failure varied with the timing of sampling, samples were grouped according to the month in which the pre-treatment samples was collected. Contingency tables were created for month of sampling and treatment failure for each anthelmintic drug class separately and for all classes combined. Contingency tables were used to perform *χ*^2^ tests with P < 0.05 considered significant.

## Results and discussion

### Response to STAP drench task

A total of 1,893 sheep producers participated in the drench test task as part of qualification in STAP. Samples were submitted to the labs between early June and early October. The distribution of participants by county is shown in Table 1. A number of farmers (n = 308) failed to provide both pre- and post-treatment faecal samples and were excluded from the study. From the remaining 1,585 participants, 82.5% recorded the name of the anthelmintic used. BZ was the most popular class of anthelmintic, used by 46.3% of participants. LEV and ML were used by 23.2% and 28% of participants respectively. A small proportion of participants (2.4%) used a combination (BZ/LEV) anthelmintic or a flukicide only product and were excluded from the study. Participants were also excluded from the study if the dates of the pre- and post-treatment sample collection were not reported or if the interval between sample collection was inappropriate for the anthelmintic product used. A total of 527 farmers (27.8%) complied with the instructions for the drench test. Reasons for data exclusion are summarised in Table 2. Fifteen additional tests were excluded from the study as the FEC testing laboratory failed the proficiency testing. Where pre-treatment flock FEC was ≤200 eggs per gram, farms were also excluded. This was the case for 143 and 410 samples for trichostrongyle FEC and *Nematodirus* FEC respectively.

**Table 1:** Distribution of samples by county

**County**	**BZ**	**LEV**	**ML**	Other/unknown*	**Total no. of samples**
Carlow	17	6	15	19	57
Cavan	15	4	4	12	35
Clare	1	1	1	1	4
Cork	20	6	10	28	64
Donegal	165	75	104	102	446
Dublin	10	1	4	3	18
Galway	88	42	42	70	242
Kerry	25	25	27	20	97
Kildare	4	8	7	6	25
Kilkenny	6	6	10	9	31
Laois	4	1	12	4	21
Leitrim	12	8	9	7	36
Limerick	1	0	0	1	2
Longford	5	4	1	1	11
Louth	8	5	4	1	18
Mayo	102	34	40	46	222
Meath	27	10	18	13	68
Monaghan	11	8	6	5	30
Offaly	3	4	8	6	21
Roscommon	40	18	14	11	83
Sligo	33	22	17	8	80
Tipperary	11	2	15	25	53
Waterford	2	8	2	5	17
Westmeath	29	8	13	8	58
Wexford	21	13	18	5	57
Wicklow	31	18	18	13	80
Unknown	1	1	5	10	17

*Other includes products only active as flukicides and combination anthelmintics with both BZ and LEV.

**Table 2:** Details of response to STAP drench test task

**Information**	**n**	**Percentage**
Total number of participants	1893	100
One faecal sample provided	308	16.3
Two faecal samples provided	1585	83.7
***Anthelmintic***		
Information on product missing	277	14.6
Ineligible product used	32	1.7
Information on sampling times missing	130	6.9
Interval pre- and post-sampling incorrect	619	32.7
**Total who complied with instructions**	**527**	**27.8**
***FEC***_***OT***_		
FEC_OT_ pre sampling ≤ 200 epg	143	7.6
Lab failed proficiency test	15	0.8
**FEC**_**OT **_**pre sampling ≥200 epg**	**369**	**19.5**
***FEC***_***NEM***_		
FEC_NEM_ pre sampling ≤ 200 epg	410	21.7
Lab failed proficiency test	2	0.11
**FEC**_**NEM **_**pre sampling ≥200 epg**	**115**	**6.1**

Percentage represents proportion from the total number of participants (n = 1,893) who participated in the drench task as part of STAP.

### Treatment efficacy against trichostrongyles

**Figure 1:**
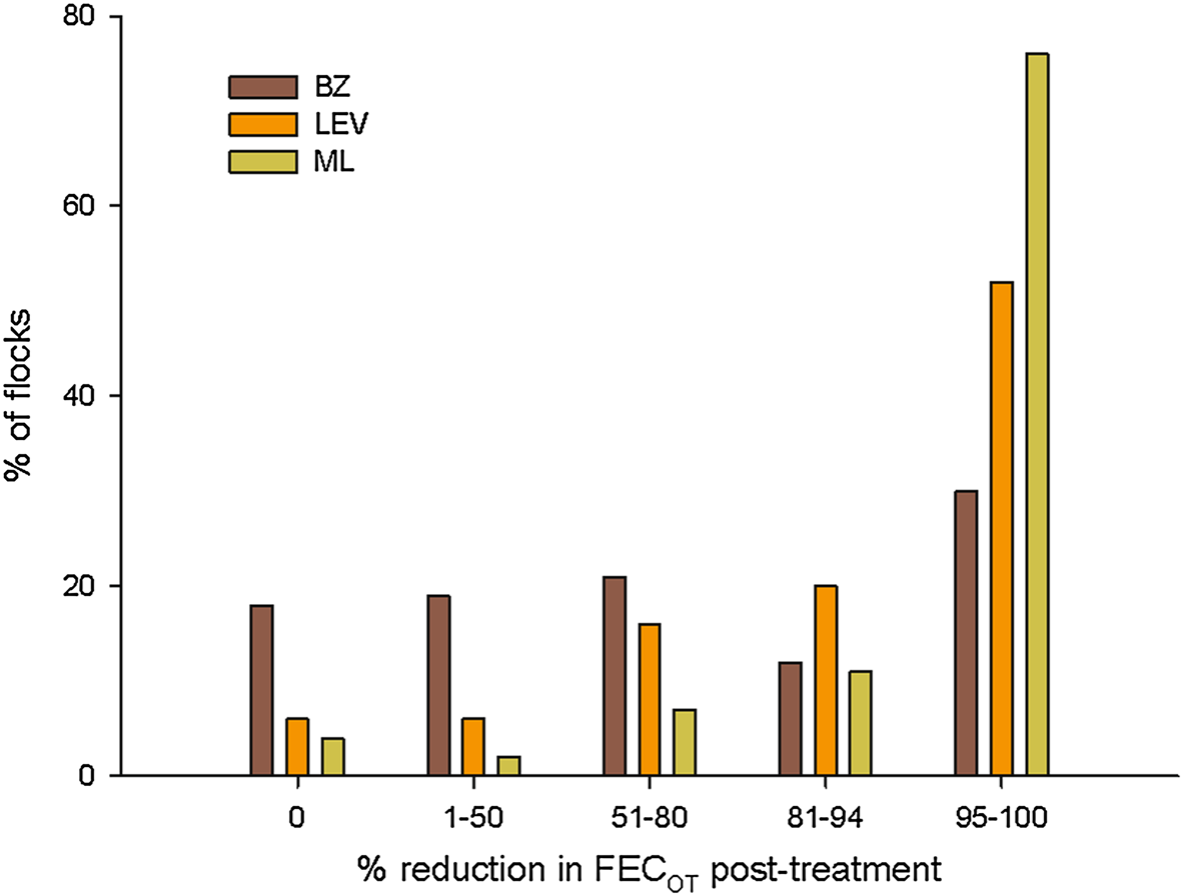
**Flocks classified according to percentage of egg reduction in FEC**_**OT **_**post-treatment with BZ, LEV or ML.**

The efficacy of BZ (n = 155), LEV (n = 82) and ML (n = 132) treatments are shown in Figure 1. There was a significant difference in the efficacy of the three drug classes (*χ*^2^ = 58.96; P < 0.0001). BZ was the least efficacious treatment as only 30% (n = 47) of BZ treatments were effective. This was followed by LEV with 52% (n = 43) efficacy. ML treatments were the most efficacious providing a ≥95% reduction in trichostrongyle FEC in 76% (n = 100) of cases. There was no relationship between treatment failure and either geographical location or the month of sampling. Nine farmers repeated the task, four using the same product as the original test and five switching to a new active ingredient. For those that repeated the task with the same anthelmintic class the results from both tests agreed in two cases, however in the other two cases the results did not agree.

### Treatment efficacy against Nematodirus

**Figure 2:**
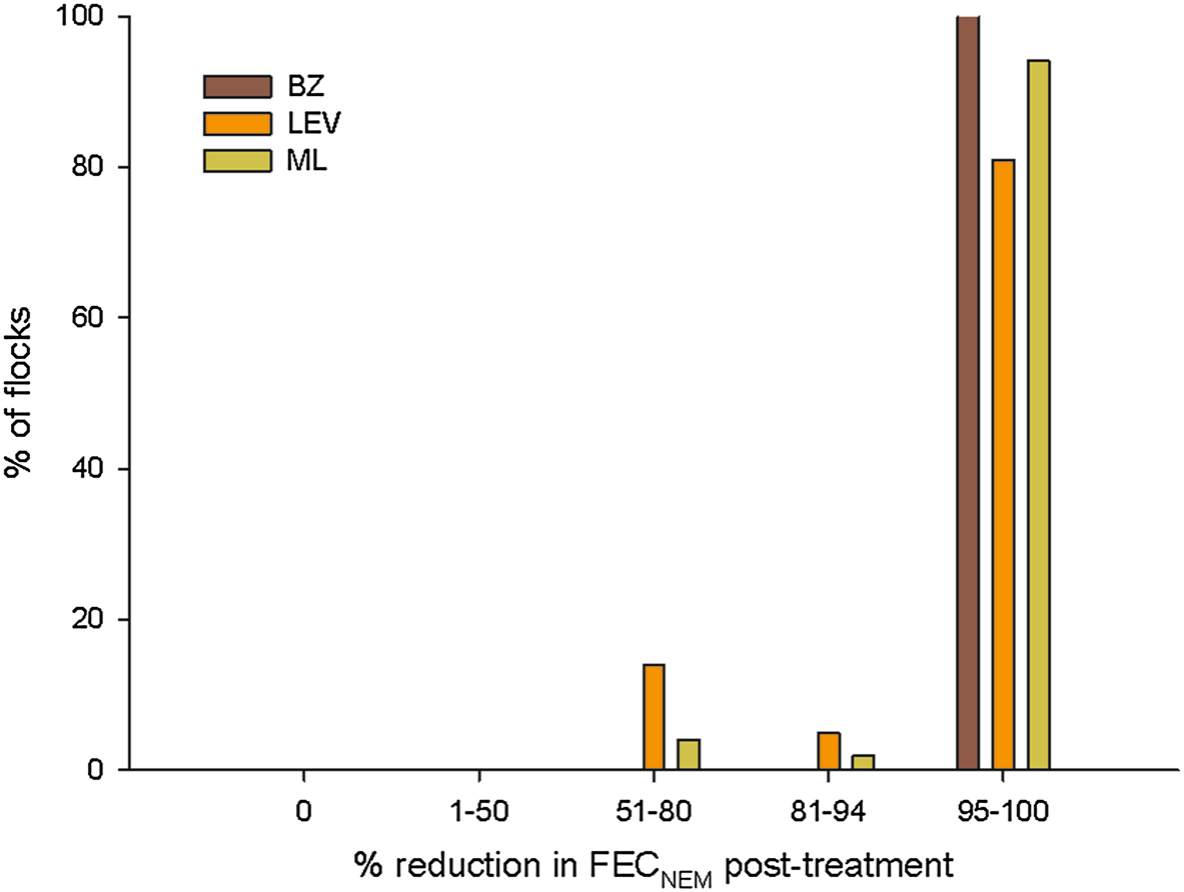
**Flocks classified according to percentage egg reduction in FEC**_**NEM **_**post-treatment with BZ, LEV or ML.**

The efficacy of BZ (n = 48), LEV (n = 20) and ML (n = 47) treatment against *Nematodirus* species are shown in Figure 2. BZ treatment was effective in 100% of tests. LEV treatment was effective in 80% of tests while in 94% of cases the ML treatment was effective against *Nematodirus* species.

With almost 2,000 farmers opting to complete the drench test task as one of their two technical tasks for inclusion in the STAP programme it became the most popular task. Despite this encouraging response, compliance with task instructions was low (27.8% n = 527). While, 16% of participants submitted only one sample, almost 15% of farmers did not report which anthelmintic product they used and the efficacy of these treatments could not be included in the study. Approximately 2% of farmers used an inappropriate product, such as a product active only as a flukicide or a combination (BZ/LEV) anthelmintic, and were also excluded from the study. A further 39.6% of farmers either did not provide information on time of sampling or did not meet the criteria concerning timeframes pre- and post-treatment (10–14, 4–7 or 14–18 days for BZ, LEV and ML products, respectively).

Over 1300 farmers reported which anthelmintic product they used and amongst those farmers 46% used a BZ product, making this drug class the most popular choice. Resistance to BZ among nematode populations on Irish sheep farms was first reported in 1992 [17] and has been widely reported since [2,5,10,18]. More recent studies have found that over 80% of flocks tested in Ireland have shown evidence of resistance to BZ [2,5]. Despite these reports, BZ was still the most popular anthelmintic product choice indicating that farmers are failing to take on board the reports of widespread resistance to BZ products. Farmers in this study were not asked to list the reason for their choice of anthelmintic product. However, a recent study found that past experience and advice from their veterinary practitioner or agricultural advisor were the main factors influencing anthelmintic product choice [13]. This represents an opportunity to disseminate awareness of the high failure rate of BZ products among the sheep farming community through these channels. ML products were the next most popular product choice. While resistance to ML products has been reported in other countries and in Northern Ireland [4-7], until recently resistance to these products among sheep nematode populations in the Republic of Ireland has only been suspected [2]. However, in 2013 ivermectin resistant *Teladorsagia circumcincta* was identified on an Irish farm (unpublished data). Of the three commonly used anthelmintic drug classes, LEV products were the least popular choice among STAP participants. Resistance to LEV has been reported on almost 40% of farms in the Republic of Ireland [2]. Many participants (n = 176) reported using a wormer/flukicide combination product.

The STAP drench test task was designed to determine if anthelmintic treatment, as practiced on Irish farms, was effective. In this study we found that approximately 51% of treatments were deemed to be effective according to the World Association for the Advancement of Veterinary Parasitology (WAAVP) guidelines (≥95% reduction in faecal egg count). This task was not designed to provide information on why treatment failed. There are many reasons why anthelmintic treatment may fail, including incorrectly estimating the dose rate, inaccurate dosing technique or faulty dosing equipment, incorrect product use or storage or the presence of anthelmintic resistance among the nematode population targeted. However, there was a significant difference between the treatment efficacies of the three commonly used drug classes. The level of treatment success was lowest with BZ products (30%) and highest with ML products (76%). These results are in agreement with previous work on anthelmintic resistance carried out in Ireland [2] which indicated levels of susceptibility to each of the drug classes as 39%, 72% and 89% for BZ, LEV and ML products respectively. Therefore treatment failure may indicate the presence of an anthelmintic resistant nematode population in many cases.

The efficacy of *Nematodirus* species treatment with the commonly used broad spectrum anthelmintics was also evaluated, 94% of treatments administered were effective against *Nematodirus* infection. Worthy of note is that BZ treatment was efficacious in all samples examined. For LEV and ML, four and three tests respectively indicated treatment failure. There have been only a few reports of anthelmintic resistance among *Nematodirus* species in Ireland or Britain [19,20] and our study is in agreement with these findings. The reason for *Nematodirus* treatment failure is unknown but it may represent true anthelmintic resistance, although further work would be required to determine this. However, *Nematodirus spp* are considered to be dose-limiting species for ML treatment and this may be one of the reasons for lower efficacy of ML in some cases [21,22]. Additionally, given the small number of cases (n = 7) combined with the fact that BZ treatment was 100% efficacious, suboptimal dosing cannot be excluded as a reason for treatment failure.

## Conclusions

Only 51% of anthelmintic treatments administered by farmers who completed a drench task as part of the STAP programme were fully effective. The reason for treatment failure is currently unknown. However, there was a significant difference in treatment efficacies with different classes of anthelmintics for trichostrongyles and the vast majority of treatments were effective for *Nematodirus* species, a parasitic species for which anthelmintic resistance is rarely reported. This implies that anthelmintic resistance is likely to be responsible for many of the cases of treatment failure. However, considering previous work on the approach of sheep producers to treatment [13], failure for reasons other than anthelmintic resistance are also possible. Irrespective of the reason for treatment failure, this study provides strong evidence that anthelmintic treatment as practiced in Irish sheep flocks has a high failure rate and needs to be addressed.

## Consent

Written informed consent was obtained from the farmers for the publication of this report and any accompanying images.

## Abbreviations

BZ: Benzimidazole; LEV: Levamisole; ML: Macrocyclic lactone; FEC: Faecal egg count; DAFM: Department of Agriculture Food and the Marine; STAP: Sheep Technology Adoption Programme; FEC_OT_: faecal egg count for other trichostrongyles (excluding *Strongyloides papillosus*); FEC_NEM_: Faecal egg count for *Nematodirus* species; WAAVP: World Association for the Advancement of Veterinary Parasitology.

## Competing interests

The authors’ declare that they have no competing interests.

## Authors’ contributions

MS coordinated the project. OMK, BG, TDW, JF, MG, MC and MS contributed to the study design. CH and MS participated in sample collection and FEC validation. OMK, JDK, CH and MS performed sample collation and analysis. All authors participated in manuscript preparation and read and approved the final manuscript.

## Authors’ information

OMK: B.A.(mod), P.Grad.Dip., Ph.D., Senior Researcher Teagasc. JDK: B.A.(mod), M.Sc, Ph.D. student Teagasc, DAFM and UCD. BG: B.A.(mod), P.Grad.Dip., Ph.D., Senior Researcher in Parasitology, Teagasc. TDW: B.V.Sc., Ph.D., DipDatMet, HDipUTL, DipEVPC, MRCVS, Senior Lecturer and European Veterinary Specialist in Parasitology, UCD. JF: B.V.Sc., Ph.D., Research Officer, DAFM. MG: B.Ag.Sc., M.Ag.Sc. Head of Sheep Knowledge Transfer, Teagasc. MC: B.V.Sc., Ph.D, Head of Regional Veterinary Laboratory Division, DAFM. CH: Kilkenny RVL. MS: B.V.Sc., Research Officer, DAFM.
